# Retraction Note: Proteogenomics of diffuse gliomas reveal molecular subtypes associated with specific therapeutic targets and immune-evasion mechanisms

**DOI:** 10.1038/s41467-026-76428-0

**Published:** 2026-08-06

**Authors:** Yunzhi Wang, Rongkui Luo, Xuan Zhang, Hang Xiang, Bing Yang, Jinwen Feng, Mengjie Deng, Peng Ran, Akesu Sujie, Fan Zhang, Jiajun Zhu, Subei Tan, Tao Xie, Pin Chen, Zixiang Yu, Yan Li, Dongxian Jiang, Xiaobiao Zhang, Jian-Yuan Zhao, Yingyong Hou, Chen Ding

**Affiliations:** 1 State Key Laboratory of Genetic Engineering and Collaborative Innovation Center for Genetics and Development, School of Life Sciences, Institute of Biomedical Sciences, Human Phenome Institute, Zhongshan Hospital, Fudan University, Shanghai, 200433 China; 2 Department of pathology, Zhongshan Hospital, Fudan University, Shanghai, China; 3 Department of Urology, Fudan University Shanghai Cancer Center, Shanghai, China; 4 Shanghai Genitourinary Cancer Institute, Fudan University, Shanghai, China; 5 Department of Neurosurgery, Zhongshan Hospital, Fudan University, Shanghai, China; 6 Institute for Development and Regenerative Cardiovascular Medicine, MOE-Shanghai Key Laboratory of Children’s Environmental Health, Xinhua Hospital, Shanghai Jiao Tong University School of Medicine, Shanghai, 200092 China; 7 School of Basic Medical Sciences, Zhengzhou University, Zhengzhou, China

Retraction Note to: *Nature Communications* 10.1038/s41467-023-36005-1, published online 31 January 2023

The authors have retracted this article. After publication, concerns were raised regarding potential similarities between Supplementary Figs. 13F-2 and 13F-4; Supplementary Figs. 13F-9 and 17I-3; Supplementary Figs. 17I-5 and 17I-8; Supplementary Figs. 21A-7 and 21A-9; and Supplementary Figs. 21B-3 and 21B-5. The Editors therefore no longer have confidence in the reliability of the data reported in the article.

Yunzhi Wang has stated on behalf of all authors that they agree with this retraction.

